# Mechanical and Tribological Behavior of Mechanically Alloyed Ni-TiC Composites Processed via Spark Plasma Sintering

**DOI:** 10.3390/ma13225306

**Published:** 2020-11-23

**Authors:** Ganesh Walunj, Anthony Bearden, Amit Patil, Taban Larimian, Jijo Christudasjustus, Rajeev Kumar Gupta, Tushar Borkar

**Affiliations:** 1 Department of Mechanical Engineering, Washkewicz College of Engineering Cleveland State University, Cleveland, OH 44115, USA; g.walunj@vikes.csuohio.edu (G.W.); a.bearden@vikes.csuohio.edu (A.B.); a.k.patil@vikes.csuohio.edu (A.P.); t.larimian@vikes.csuohio.edu (T.L.); 2 Department of Material Science and Engineering, North Carolina State University, Raleigh, NC 27606, USA; jchris22@ncsu.edu (J.C.); rkgupta2@ncsu.edu (R.K.G.)

**Keywords:** metal matrix composites (MMCs), mechanical alloying (MA), spark plasma sintering (SPS), nickel-titanium carbide composites, microhardness

## Abstract

Titanium carbide (TiC) reinforced nickel (Ni) matrix composites were processed via mechanical alloying (MA) followed by spark plasma sintering (SPS) process. Mechanical alloying has gained special attention as a powerful non-equilibrium process for fabricating amorphous and nanocrystalline materials, whereas spark plasma sintering (SPS) is a unique technique for processing dense and near net shape bulk alloys with homogenous microstructure. TiC reinforcement varied from 5 to 50 wt.% into nickel matrix to investigate its effect on the microstructure and mechanical behavior of Ni-TiC composites. All Ni-TiC composites powder was mechanically alloyed using planetary high energy ball mill with 400 rpm and ball to powder ratio (BPR) 15:1 for 24 h. Bulk Ni-TiC composites were then sintered via SPS process at 50 MPa pressure and 900–1200 °C temperature. All Ni-TiC composites exhibited higher microhardness and compressive strength than pure nickel due to the presence of homogeneously distributed TiC particles within the nickel matrix, matrix grain refinement, and excellent interfacial bonding between nickel and TiC reinforcement. There is an increase in Ni-TiC composites microhardness with an increase in TiC reinforcement from 5 to 50 wt.%, and it reaches the maximum value of 900 HV for Ni-50TiC composites.

## 1. Introduction

Over the past few decades, metal matrix composites (MMC) have attracted considerable interest due to their exceptional physical and mechanical properties, such as high specific modulus, fatigue strength, thermal stability, and wear resistance. These outstanding properties make MMCs suitable for a wide range of applications, including aerospace and automotive industry, and other structural, electrical, and thermal applications [1]. Low coefficient of thermal expansion and higher thermal conductivity that makes them ideal for use in electronic applications. MMCs are broadly classified into two types based on the reinforcement structure within the base metal matrix. One of the types of reinforcement is continuous reinforcement, which includes fibers and sheets of either a metal or composite reinforcement [2]. The other type is discontinuous reinforcement, that includes particles, short fibers or whiskers, and other small particles like structures [3]. Discontinuous reinforcement has several advantages over continuous reinforcement by avoiding nonuniformity and fiber to fiber contact [3].

While a lot of work has been done in the area of iron (Fe) and aluminum (Al) based MMCs, there has not been much work in the field of nickel (Ni) based MMCs, although there has been a significant increase in the interest surrounding them. Although Ni is a relatively soft metal that may not appear optimal for applications where high strength is required, it exhibits excellent bonding with harder reinforcement and substrates [4,5], making it an ideal candidate as a base material for MMCs. Since Ni is a soft and ductile metal, it needs to be reinforced with a much harder material such as TiC, TiN, SiC, etc., to enhance its strength and to be used in practical structural applications. TiC has been widely used as a reinforcement in MMCs due to its high wear resistance [6], high melting point, low density, and excellent creep resistance. One of the limitations of using TiC alone in structural applications is that the very high hardness of the material (2859–3200 HV) [7] makes it very brittle and prone to cracking if a thermal or mechanical shock is applied. The reinforcing of TiC in the Ni matrix develops a composite that exhibits excellent resistance to cracks and wears while enhancing hardness beyond pure nickel. Therefore, titanium carbide reinforced nickel matrix composites are considered potential candidates for high-temperature structural applications. Additionally, TiC reinforced Ni matrix composites exhibit excellent interfacial bonding due to the low wetting angle between nickel and titanium carbide. Overall, TiC reinforced Ni matrix composites exhibit the right balance of properties combining high strength and hardness of the TiC with the nickel’s ductility and toughness, making them ideal candidates for structural applications. Ni-TiC composites have many applications where the ability to perform under high temperature with a high strength to mass ratio is required. These Ni-TiC composites can be used for structural applications in the aerospace, automotive, and defense industries. 

Mechanical alloying is a method of dry alloying powders, which was invented in 1966 and rapidly gained popularity as a novel method of producing alloyed and composite powders [8]. The milling tool has a vital role in the mechanical alloying process. The ball mill uses particle mechanochemistry that provides chemical reaction from mechanical activities, a similar methodology used in the tumbling mills, invented in the 1870s for industrial mining and aggregate production applications [8]. The tumbling mill’s fundamental issue is that the milling energy is limited to the acceleration of the milling media due to gravity [9]. That limitation was overcome with the development of the planetary ball mill by Fritsch GmbH in 1961 [10], which utilizes centrifugal force to accelerate the milling medium, allowing milling energies much higher than that of the tumbling mill. The planetary ball mill became the workhorse for laboratory work because of the wide variety of sample types it can process and the ability to do dry and wet milling [10]. One of the important considerations when using ball milling for alloying and mixing is the prevention of cold welding due to high energies heat generation during the milling process. Most commonly, a Process Control Agent (PCA) is added to the mixture to be milled to prevent excessive cold welding of the milled powders and can also help in improving dispersion [1,11,12]. Another consideration of ball milling is the milling process time and intensity; as time increases, the contamination level from wear of the milling surfaces increases, which would create undesired phases in the material [13]. All milling parameters such as milling duration, speed, and ball to powder ratio (BPR) significantly affect the alloying process. The milling process duration should be such that the powder particle reaches a uniform size through the rate of fracture, and cold welding reaches an equilibrium.

The concept of current assisted sintering is described in several patents issued as early as 1933. Since then, the process has been developed with slight variations going by different names such as Spark Plasma Sintering (SPS), Plasma-Assisted Sintering (PAS) [14]. Part of the reason for these different names of the same process is that there are conflicting results regarding the existence of plasma or sparks in the SPS process [15,16,17,18,19]. The evidence for the existence of plasma in the sintering process is insufficient to prove its existence, while the evidence showing the lack of sparks or plasma is far more convincing [12]. Many researchers choose to refer to the process by names they believe are a correct description of the process. In the SPS process, the sample is placed in a conductive die and pressed using conductive punches, which allow large amounts of current to pass through the sample, heating it to the desired temperature [20]. One advantage of SPS is that it can sinter at comparatively lower temperatures and pressures than other methods such as hot pressing [21]. Another advantage of the SPS process is that it can heat the sample quickly through a mechanism known as resistance pulse heating, also known as Joule heating [12,20,22]. Being able to heat the sample quickly shortens the sintering time, which translates to smaller grain sizes than cannot be achieved with other solid-state pressing methods [12]. Spark plasma sintering is a novel tool for processing metals, alloys, and composites at lower temperatures and shorter processing times than conventional processing routes. Mechanical alloying (MA) has gained special attention as a powerful non-equilibrium process for processing alloys and composite powders, whereas spark plasma sintering (SPS) is a unique technique for processing dense and near net shape bulk materials with homogenous microstructure. Mechanical alloying, combined with SPS processing, will open up new avenues of processing near-net-shape components of metals, alloys, and metal matrix composites.

The development and production of nickel-based composites have been studied before. However, the production methods used to create these composites significantly influence the final properties of the components themselves. In the present investigation, Ni-TiC composites have been processed via mechanical alloying followed by SPS processing. There are very limited studies that have been done on understanding the effect of TiC content on microstructure and mechanical properties of Ni-TiC composites. This investigation’s primary objective is to study the effect of TiC content on microstructure, mechanical, and tribological behavior of SPS processed Ni-TiC composites. The present study will form the basis for the systemic selection of TiC content for the processing of Ni-TiC composites with target mechanical and tribological properties.

## 2. Materials and Methods

### 2.1. Mechanical Alloying

The Ni-TiC composite powder was prepared from an elemental mixture of Ni metal powder (particle size~ 3–7 µm, purity > 99.9%, Alfa Aeser, Haverhill, MA, USA) and TiC metal powder (particle size~ 7–10 µm, purity > 99%, Alfa Aeser) powders alloyed via high energy ball mill (Pulverisette 7 Premium, Fritsch, Germany). The mechanical alloying has been carried out for 24 h in a tungsten carbide 80 mL grinding bowl and 3 mm size balls with a ball to powder weight ratio (BPR) of 10:1 and 300 rpm. The milling operation carried in two steps involves 20 min of milling and 10mins of the cooling interval to avoid overheating the sample. Steric acid (2 wt.%) was used as a process control agent (PCA) during mechanical alloying to prevent excessive cold welding due to high energy generated during the alloying process. The TiC content inside composites has been systematically increased from 5 to 50 wt.% to investigate its effect on these composites’ microstructure and mechanical properties.

### 2.2. Spark Plasma Sintering

The Ni-TiC composite powders were pre-compacted in a graphite mold under a pressure of 5 MPa. The pre-compacted powders were sintered by SPS system (SPS 10-3, Thermal Technology LLC, Santa Rosa, CA, USA) at a temperature of 900–1200 °C for 5 min under a controlled argon atmosphere under a pressure of 65 MPa. The SPS processing parameters for pure nickel and Ni-TiC composites are listed in Table 1. All sintered samples exhibit 20 mm diameter and 3 mm thickness. All samples were cooled to room temperature; after that, they were ground to remove graphite foils attached to them during the sintering process and then mounted using conductive mold material. Mounting samples were ground (AutoMet 250, Buehler, IL, USA) using 240, 400, 600, 800, and 1200 grit sandpaper sequentially. Finally, samples were polished using colloidal silica to achieve a smooth surface. The samples were then ultrasonically cleaned in DI water, soap solution, and finally in ethanol to remove the silica from the surface.

### 2.3. Scanning Electron Microscopy and X-ray Diffraction

The microstructures of all samples were analyzed using Scanning Electron Microscopy (SEM) (Inspect F50, Manufacturer: FEI now ThermoFisher Scientific, Hilsboro, OR, USA). Grain sizes were measured using ImageJ software, and an average of 50 grains were reported in this paper. The phase analysis of all sintered samples was performed using an X-ray diffraction (1.54 Cu Kα) line of a Rigaku Ultima III x-ray diffractometer. 

### 2.4. Microhardness, Compression, and Tribological Testings

The SPS-processed samples microhardness measurements were carried using a standard Vickers microhardness tester (Wilson, Chicago, IL, USA) under a load of 5 N for 10 s. The average of those ten readings is used as the hardness for each sample. The cylindrical specimens with a 3 mm diameter and a height of 6 mm were machined using a wire-EDM machine and then used for the compression tests. The loading surface was polished parallel to the other, and lubrication oil was applied on both surfaces to avoid a barreling effect due to friction. The compression tests were carried out on the Instron 3369 universal testing machine with a strain rate of 1 × 10^−4^ s^−1^. Dry (unlubricated) wear tests were performed on a ball-on disc tribometer (Nanovea™, Irvin, CA, USA) at room temperature and the standard test method for wear testing pin on disc apparatus has been followed [23]. Silicon nitride (Si_3_N_4_) ball (diameter 6 mm) was used as a counter body. The wear tests were conducted in the dry atmosphere (relative humidity~50%) using a load of 1 N, sliding velocity of 200 rpm, and track diameter of 5 mm (total sliding distance = 140 m). EDS elemental analysis using SEM was used for a better understanding of wear mechanism and track study.

## 3. Results and Discussion

### 3.1. XRD Testing

**Figure 1:**
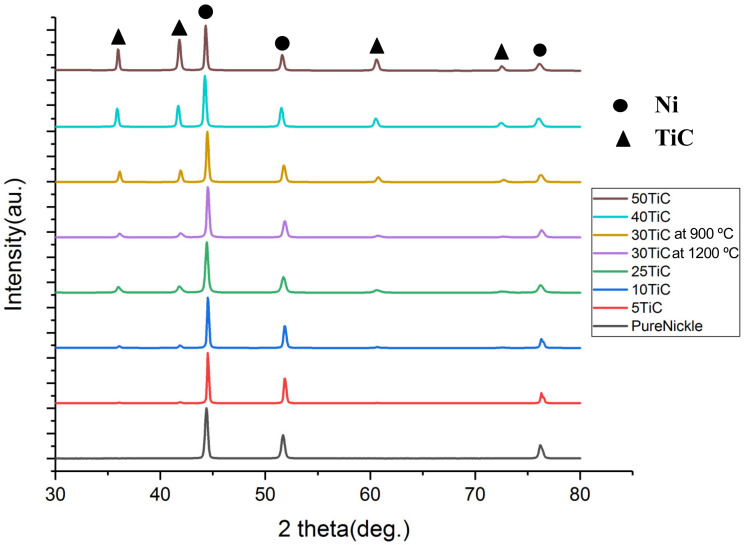
XRD graph and peak index of Pure Ni, Ni-5TiC, Ni-10TiC, Ni-25TiC, Ni-30TiC at 900 °C, Ni-30TiC at 1200 °C, Ni-40TiC and Ni-50TiC.

Figure 1 illustrates the x-ray diffraction (XRD) patterns for SPS processed pure nickel and Ni-TiC composites. The XRD pattern of pure nickel exhibits peaks correspond to the (111), (200), and (220) crystallographic planes of FCC-nickel. Furthermore, XRD patterns of all Ni-TiC composites exhibit peaks correspond to the rocksalt NaCl-type TiC phase in addition to the FCC-nickel phase. As TiC content increases in Ni-TiC composites from 5 wt.% to 50 wt.%, the intensity of TiC peaks increases and became very dominant in Ni-50 wt.% TiC composites. In all XRD patterns obtained from pure nickel and Ni-TiC composites, FCC Ni (111) peaks exhibit the highest intensity. The Ni(111)/Ni(200) intensity ratio for SPS processed pure Ni is 2.15; while that for Ni-TiC composites Ni-5TiC, 2.03; Ni-10TiC, 2.30; Ni-25TiC, 3.24; Ni-30TiC 900 °C, 3.08; Ni-30TiC 1200 °C, 3.03; Ni-40TiC, 2.83 and finally for Ni-50TiC, 2.45. All these ratio values are in good agreement with the standard ratio expected for randomly oriented Ni grains of 2.38 (based on the International Center for Diffraction Data (ICDD) files obtained from the Joint Committee of Powder Diffraction Standards (JCPDS)). No significant change in texture results from the introduction of TiC in the nickel matrix. No intermetallic peaks correspond to Ni-Ti, or Ni3Ti observed in these XRD patterns, confirming the absence of any in situ reactions between pure nickel and TiC during mechanical alloying as well as during SPS processing. Additionally, TiC is thermodynamically stable than Ni_3_Ti, which cannot hold high sintering temperatures [7]. 

### 3.2. Scanning Electron Microscopy (SEM) Analysis and Relative Density

**Figure 2:**
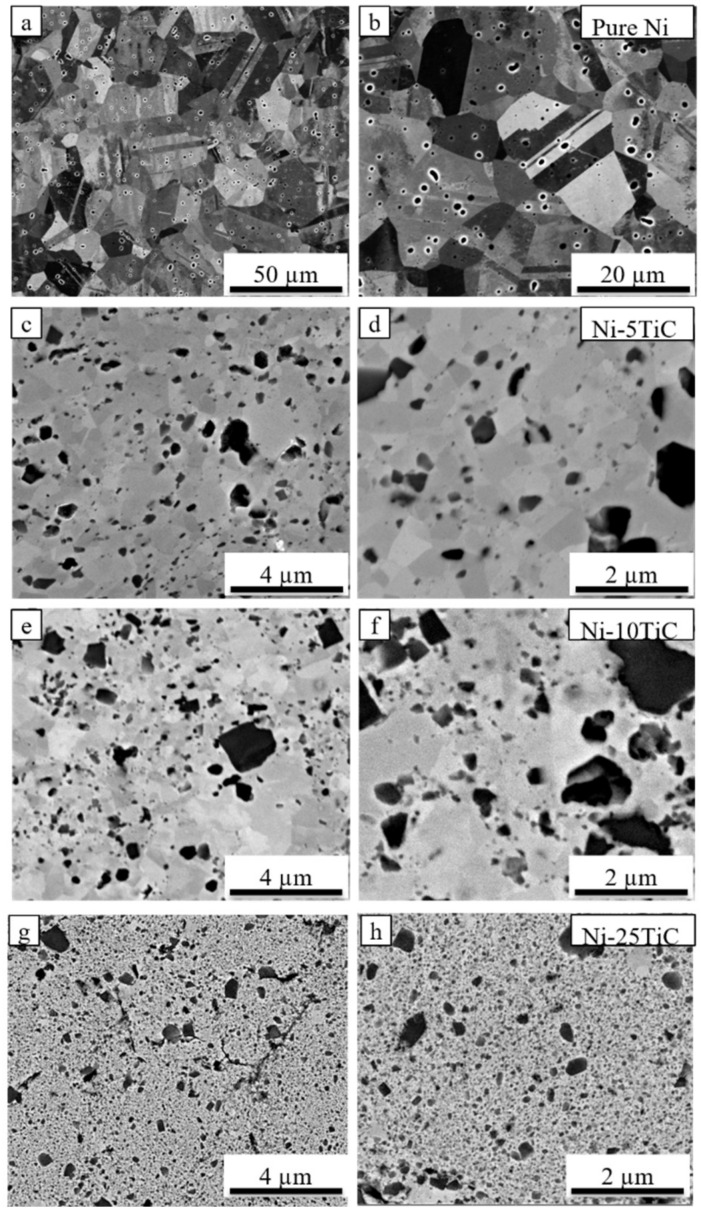
Scanning electron microscopy images of spark plasma sintered (**a**,**b**) Pure Ni, (**c**,**d**) Ni-5TiC, (**e**,**f**) Ni-10TiC, and (**g**,**h**) Ni-25TiC.

**Figure 3:**
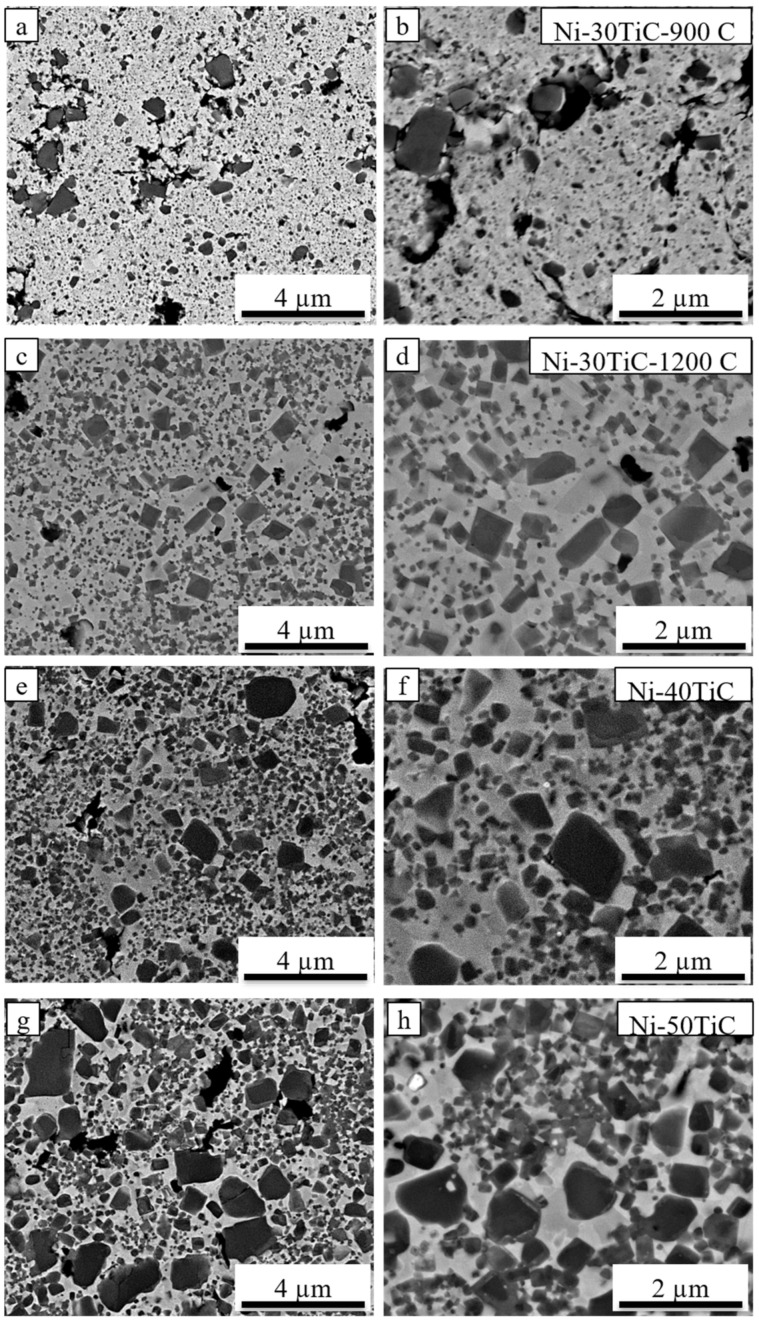
Scanning electron microscopy images (**a**,**b**) Ni-30TiC at 900 °C, (**c**,**d**) Ni-30TiC at 1200 °C, (**e**,**f**) Ni-40TiC, and (**g**,**h**) Ni-50TiC Composites.

**Figure 4:**
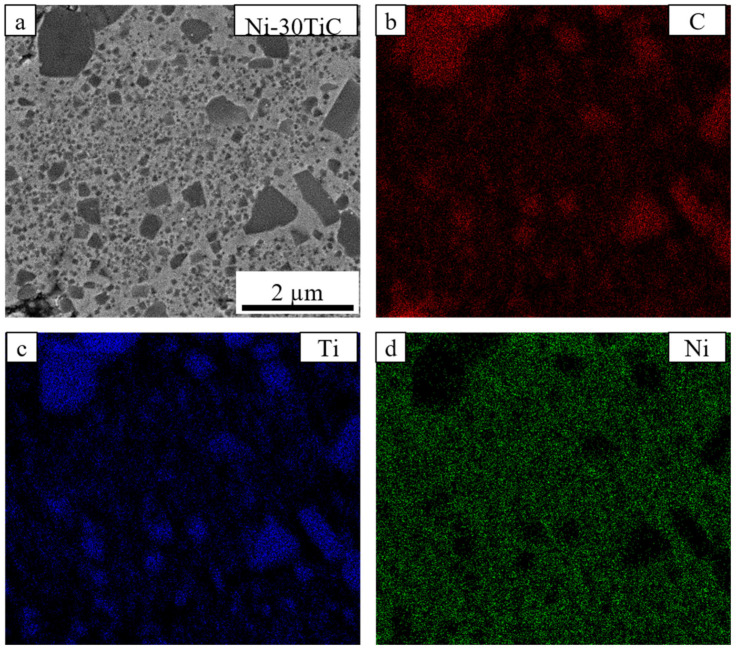
(**a**) Scanning electron microscope image and EDS elemental analysis of (**b**) Carbon, (**c**) Titanium, and (**e**) Nickel of Ni-30TiC-1200 °C composite.

Figure 2 and Figure 3 shows backscattered SEM images of spark plasma sintered pure nickel and Ni-TiC samples with varying wt.% of TiC reinforcement. Pure nickel exhibits a uniform grain structure with 98.2% relative density and average grain size of 33 μm. The full and rapid densification of powder compacts without any substantial grain growth is one of the major advantages of SPS processing that is a result of sintering by joule heating and the spark plasma generated by the pulsed, high electric current passing through the compact. All Ni-TiC composites (Figure 2c–f and Figure 3) exhibits the TiC phase (black particles) in addition to the grey nickel matrix. All TiC particles are uniformly distributed within the nickel matrix. Figure 4 shows the SEM-EDS map of Ni-30TiC, where black particles are enriched in Ti and C and depleted in Ni, which corresponds to the TiC phase, which is consistent with XRD results. All TiC particles are uniformly distributed within the nickel matrix in all Ni-TiC composites. Mechanical alloying significantly helped minimize TiC particle sizes since particle sizes are very small in SPS processed Ni-TiC composites compared to starting particle size. SEM images clearly showing an increase in the volume fraction of the TiC phase with increasing TiC wt.% in these Ni-Tic composites. Additionally, with increasing TiC wt.%, there is a decrease in grain sizes of pure nickel since increasing volume fraction of TiC leads to an increase in the number of nucleation sites for pure nickel during recrystallization. It has also been observed that Ni-30TiC sintered at 900 °C has a smaller grain size of pure nickel as well as TiC with some porosity as compared to the sample sintered at 1200 °C. The higher sintering temperature leads to the coarsening of both nickel and TiC sizes but improves densification. Figure 4a–d shows the SEM image of Ni-30TiC sintered at 900 °C and 1200 °C, due to lack of sintering temperature, the Ni-30TiC-900 °C sample hasn’t sintered properly and exhibits porosity, compared to the Ni-30TiC-1200 °C sample that sintered properly without any porosity. Therefore, it has been found that with increasing TiC content in these composites, sintering temperature should increase for better consolidation. Thus, all samples with TiC content higher than 30 wt.% have been sintered with 1200 °C.

Table 1 illustrate the grain sizes of nickel in all SPS processed Ni-TiC composites. Pure nickel exhibited a grain size of around 33 μm. The nickel’s grain size decreases from 0.43 μm to approximately 0.19 μm as TiC content increases from 5 to 50 wt.% in Ni-TiC composites. The grain size of a nickel in Ni-TiC composites sintered at 900 °C decreases from 0.43 μm to 0.11 μm as TiC content increases from 5 to 30 wt.%. We have observed a slight increase (from 0.11 to 0.21 μm) in nickel grain size in Ni-30TiC composites sintered at 1200 °C as compared to that of sintered at 900 °C, primary due to coarsening of nickel grains at higher sintering temperature. The temperature has a significant impact on the grain size of a nickel, and it increases with increasing the sintering temperature [24]. TiC content increases from 30 to 50 wt.%, a minor decrease in the grain size have been observed from 0.21 to 0.19 μm. The relative densities of Ni-TiC composites are listed in Table 1. In the case of Ni-TIC composites sintered at 900 °C, as TiC content increases from 5 to 30 wt.%, relative density decreases from 96% (Ni-5TiC) to 84% (Ni-30TiC). However, Ni-30TiC composites sintered at 1200° C exhibited around 98% relative density. This clearly shows that in SPS processing temperature plays an important role in governing the densification behavior of Ni-TiC composites. Therefore. Optimum selection of SPS temperature is important to achieve higher density in Ni-TiC composites, particularly in a higher concentration of TiC (above 30%). The Ni-30TiC sintered at 1200 °C exhibited better microhardness, young modulus, and grain size refinement than all other Ni-TiC composites.

### 3.3. Mechanical Properties

**Figure 5:**
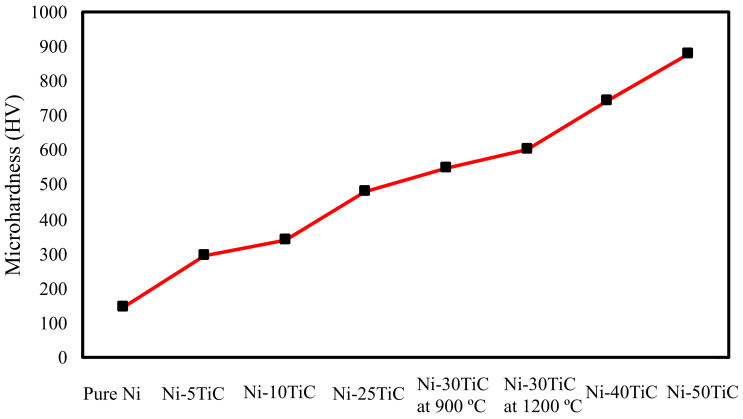
Microhardness of Pure Nickel and Ni-TiC composites.

The Vickers micro-hardness values for the pure nickel and all Ni-TiC composites have been plotted in Figure 5 and also listed in Table 2. The micro-hardness of SPS Processed pure nickel is 147.2 HV, which is lower than all Ni-TiC composites. Ni-5TiC composites exhibit microhardness of approximately 300 HV, which is double compared to that of pure nickel. Therefore, it is clear that TiC reinforcements help reduce the grain size of pure nickel and significant improvements in micro-hardness. Ni-TiC composites microhardness increases very linearly with an increase in TiC content and reaches maximum value up to approximately 900 HV with the addition of 50 wt.% TiC. The Ni-30 TiC sample sintered at 900 °C shows lower microhardness than the specimen sintered at 1200 °C. The improvement in microhardness was observed due to the improvement in the samples’ densification at higher temperatures. Abderrazak et al. [25] reported that the hardness of TiC is 2700 HV after 20 hr of MA followed by consolidation using SPS processing at 1650 °C. The Vickers microhardness of MA TiC compacted using plasma-activated sintering was found to be approximately 3200 HV [26]. El-eskandarany and A. Teber et al. [26,27] stated that microhardness of TiC sintered at 16,500 C and 100 MPa using SPS processing is 2700 HV and 2450 HV with 5 min and 10mins of holding time, respectively, and drop in hardness due to coarsening of grains at higher temperatures. The increase in hardness correlating with the increase in sintering temperature is consistent with other authors that used SPS to produce TiC based composites [24,27]. 

**Figure 6:**
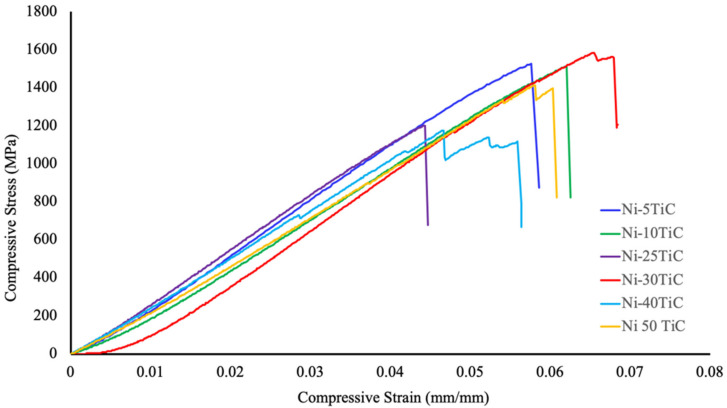
Compressive stress versus strain plots of Ni-TiC composites.

Figure 6 show compressive stress versus strain plots for Ni-TiC composites with different weight fractions of TiC, and the mechanical properties (compressive yield strength, ultimate compressive strength, and fracture strain) of the composites are summarized in Table 2. As we can see from the plot, due to TiC reinforcement’s addition, compressive strength has been significantly improved compared to pure nickel. All Ni-TiC composites exhibited almost three times higher compressive strength as compared to pure nickel. Ni-30TiC composites exhibited the highest ultimate compressive strength as compared to other Ni-TiC composites. As the weight fraction of TiC increases, the compressive strength of Ni-TiC composites increases by up to 30 wt.%. However, as TiC wt.% increases beyond 30%, for Ni-40TiC and Ni-50TiC composites, compressive properties started decreasing. This might be due to the presence of porosities in Ni-40TiC and Ni-50TiC composites sintered at 1200 °C and requires a higher temperature to achieve full densification. The authors have limited the sintering temperature to 1200 °C due to the lower melting temperature of pure nickel and to avoid any melting during SPS processing. This significant increase in strength of SPS processed Ni-TiC composites can be attributed to (a) the incorporation of TiC particles that has higher strength and modulus, (b) the grain size refinement due to the addition of TiC particles within nickel matrix, (c) increase in dislocation density to accommodate the difference between the coefficient of thermal expansion (CTE) between TiC particle and nickel matrix, and (d) thermal stability and excellent interfacial bonding between TiC particles and nickel matrix. The Ni-TiC composites exhibited significant refinement in nickel grains as compared to pure nickel due to the addition of TiC particle prevent grain growth during the sintering process (Figure 2 and Figure 3). TiC particle addition into the nickel matrix significantly increases dislocation densities in Ni-TiC composites due to the large CTE difference between TiC reinforcements and nickel matrix. Additionally, the possibility of the strengthening of nickel matrix through elastic strain resulting from CTE mismatch or due to load transfer from TiC particles to nickel matrix during deformation must be considered. TiC reinforced metal matrix composites have gained lots of attention due to the excellent thermal stability of TiC within the metal matrix and significant enhancement in hardness and strength to weight ratio of TiC reinforced MMCs. In the compressive stress-strain graph, we have observed a sudden drop in stress during compression testing, particularly in Ni-TiC composites with higher TiC wt.% (Ni-30TiC to Ni-50TiC). Few researchers observed similar behavior in ceramic reinforced composites primarily due to formation and unstable micro-crack propagation, [28,29,30]. The composites exhibited repeated crack initiation and arrest behavior, suggesting an excellent toughening effect from the interaction between the crack tip and the microstructure. These cracks bowing and deflection toughening mechanism have been proposed to increase toughness in some ceramic based systems and ceramic reinforced composites. We have also observed that the TiC wt.% and sintering temperature play an important role in governing microstructure and the resulting mechanical properties of these Ni-TiC composites. As TiC wt.% increases in Ni-TiC composites, the higher sintering temperature is required to achieve full densification and excellent mechanical properties. Therefore, Ni-TiC (5–25 wt.%) composites have been sintered at 900 °C and as TiC content increase further (30–50 wt.%), these composites have been sintered at 1200 °C.

**Figure 7:**
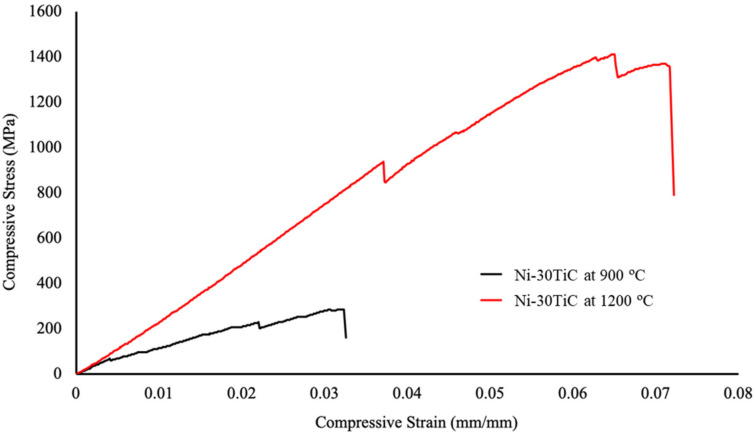
Compressive stress versus strain plots of Ni-30 TiC composites sintered via 900 °C and 1200 °C.

The compressive stress versus strain plot for Ni-30TiC composites sintered at 900 °C and 1200 °C is shown in Figure 7. The Ni-30TiC composites sintered at 1200 °C exhibited almost five times higher ultimate compressive strength as compared to Ni-30TiC composites sintered at 900 °C. This significant improvement in compressive properties is primarily due to improvement in the densification of Ni-30TiC composites sintered at 1200 °C where the load has been effectively transferred from nickel matrix to TiC reinforcement due to good interfacial bonding between TiC particles and nickel matrix. Due to porosity as well as weak interfacial bonding between TiC particles and nickel matrix, Ni-30TiC sintered at 900 °C composites exhibited very poor compressive behavior.

### 3.4. Tribological Properties

**Figure 8:**
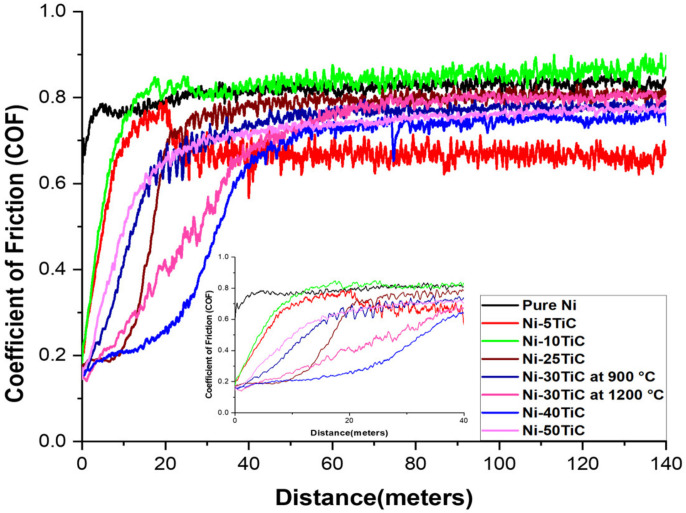
Coefficient of Friction vs. 140 m Distance.

Figure 8 shows the coefficient of friction versus rotating distance plots for the SPS processes pure nickel and Ni-TiC composites. In the tribological test, we have observed that the pure nickel has the highest COF compared to all Ni-TiC composites. The addition of 5 wt.% of TiC into the nickel matrix (Ni-5TiC) reduces COF from ~0.85 of pure nickel to ~0.65. The coefficient of friction of Ni-TiC composites increases with increasing sliding distance. At the beginning of 20 m sliding distance, the coefficient of friction (COF) comparatively low for all Ni-TiC composites (Figure 8 inset). During the dry sliding wear test, Ni-40TiC and Ni-30TiC-1200 °C exhibit ~0.2 to ~0.4 COF during the initial 20 m sliding. Dallaire and Cliché [31] studied the tribological properties of TiC-Fe coating and found that the COF value of the layer remains low until 500 m sliding distance; after that, it increases drastically due to the porosity inside the matrix causes the surface broke down. The oscillating coefficient of friction on the graph for metal matrix composite is more than pure nickel, and these wear phenomena are called the slip-line field wear mechanism [32]. As shown in Figure 5, the microhardness on Ni-TiC composites increases with increasing wt.% of TiC content into the nickel matrix. The hard composites contacting counter body during dry sliding wear testing generate high friction; simultaneously, applied load raptures particles and finally broke down the particles where the friction values go to zero [33]. This process has less adhesive wear, causing higher wear resistance without a significant difference in friction coefficient [33]. 

**Figure 9:**
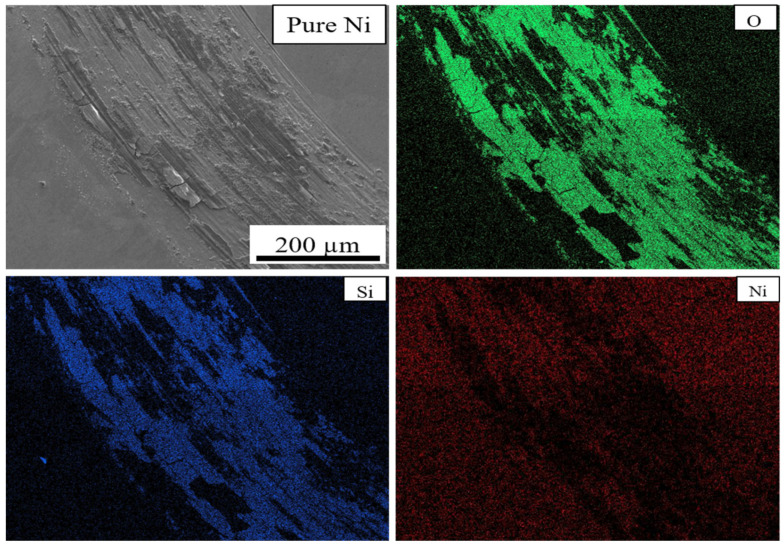
Wear Track EDS Map of Pure Ni.

**Figure 10:**
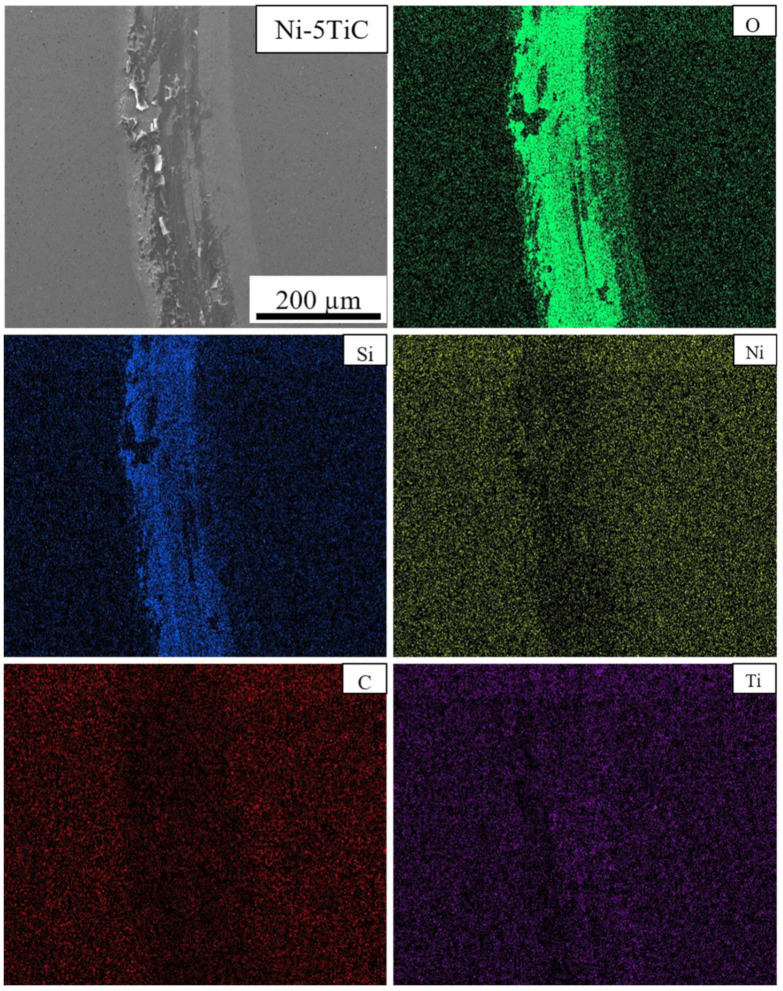
Wear Track EDS Map of Ni-5TiC.

**Figure 11:**
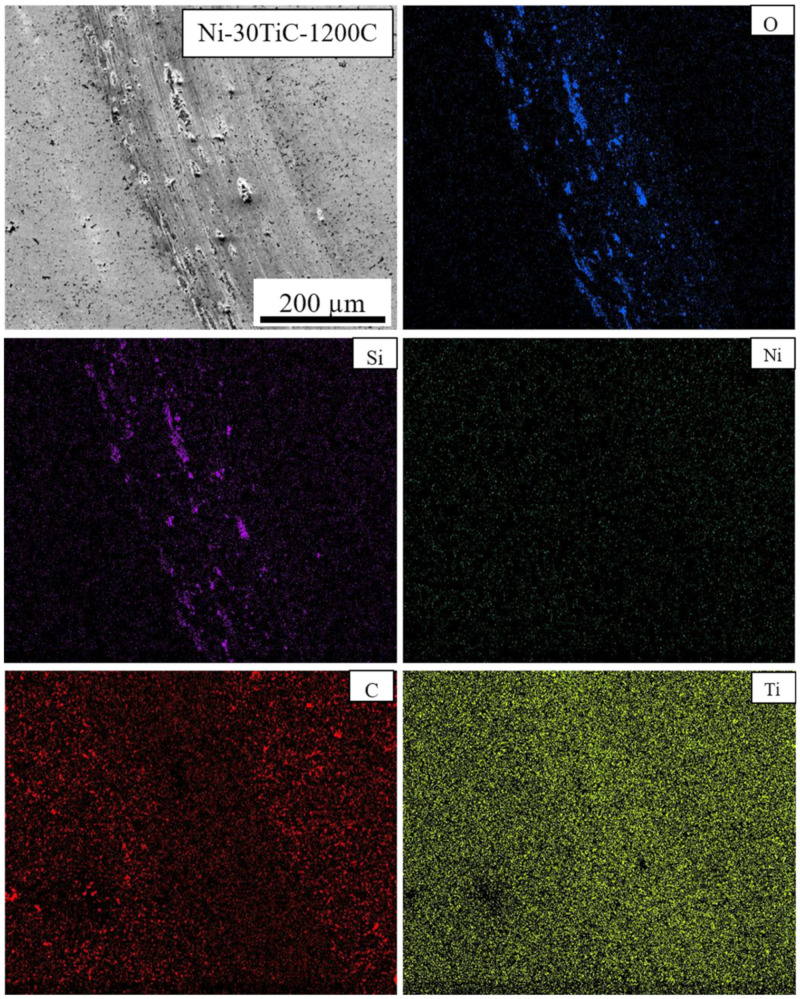
Wear Track EDS Map of Ni-30TiC-1200C.

**Figure 12:**
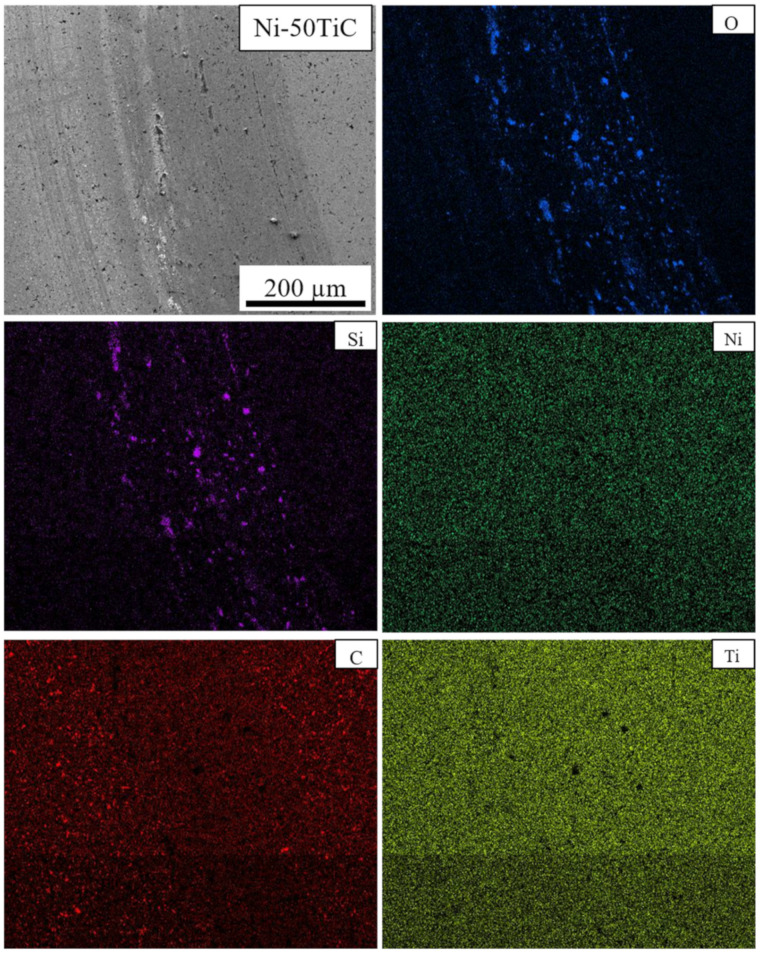
Wear Track EDS Map of Ni-50TiC.

SEM micrographs and EDS maps of the pure Ni and Ni-TiC composites after the tribotesting are illustrated in Figure 9, Figure 10, Figure 11 and Figure 12. The continuous grinding structure has been noticed throughout the track in a moving direction. The wear track consists of flaking like wear debris and scratches over the track indicating abrasive wear mechanism in Ni-TiC composites. Outside edges of the wear track exhibited wear debris; also, the Si_3_N_4_ ball wear has been observed during the wear testing. A tribolayer formed over the sliding track gives a strong indication of material transfer between the grinding surfaces. EDS map clearly shows the presence of silicon and oxygen along the wear track, indicating two to three-body abrasive wear mechanisms. In three-body wear phenomena, wear debris caught in-between the sliding surfaces, leading to the formation of tribiolayer over the sliding path; this similar mechanism is observed in the tribochemical wear. The chips and cracks and the highest silicon and oxygen presence over the sliding path revealed in EDS maps of pure nickel wear track shown in Figure 9. Nickel is a softer material and has a lower hardness result in adhesive wear of pure nickel. At the same time, an increasing weight percentage of TiC in Ni matrix reduces the chipping. As TiC content increases in Ni-TiC composites, decreases in silicon deposition on the track have been observed (Figure 10, Figure 11 and Figure 12). Due to the lower hardness of pure nickel compared to Ni-TiC composites, pure nickel exhibits the highest adhesive wear and detected deep grove and flaking. These observations show pure nickel has the highest wear rate than Ni-TiC composites. 

On the other hand, Ni-50TiC shows significantly less surface wear and minimum counter-body material detected over the track than all different samples. TiC is a harder material, and Ni-TiC composites showing less wear due to the presence of TiC increases the load-bearing capacity [34]. The flaking wear phenomena have been observed in pure nickel and Ni-5TiC samples, whereas micro ploughing wear phenomena have been observed in Ni-30TiC and Ni-50TiC composites due to the higher content of TiC in these composites. The TiC has a higher hardness than the rotating Si_3_N_4_ ball, causing a micro ploughing wear mechanism [35]. The wear resistance of Ni-TiC composites shown significant improvement than pure nickel. The friction coefficient remains approximately 0.2 to 0.4 until the initial 20 m of the sliding distance, and then it increases drastically due to high friction. The presence of TiC into the nickel matrix significantly affects the wear behavior of these composites.

## 4. Conclusions

Pure nickel and Ni-TiC composites have been successfully processed via mechanical alloying followed by spark plasma sintering process. The Ni-TiC composites exhibit 5–50 wt.% TiC reinforcements. The SPS processed pure nickel exhibit homogeneous grain structure, and the TiC reinforcements increase the number of nucleation sites, leading to refinement in pure nickel grain sizes. The refinement in grain size enhances the microhardness of Ni-TiC composites apart from microhardness improvement due to TiC reinforcement. Ni-5 wt.% TiC composites exhibit approximately two times higher microhardness than that of pure nickel. As the TiC wt.% increase in Ni-TiC composites, Ni-TiC composites microhardness increases almost linearly and reaches to the highest value of approximately 900 HV for Ni-50TiC composites. The sintering temperature plays an important role in improving the densification of these composites. The Ni-30TiC composites sintered at 1200 °C exhibits higher microhardness and densification than the sample sintered at 900 °C. Ni-30TiC composites exhibited the highest compressive strength compared to all other Ni-TiC composites primarily due to fine microstructure and stronger interfacial bonding between TiC particles and nickel matrix effectively transfer load from TiC to nickel matrix during deformation. The significant strength improvement in all Ni-TiC composites can be attributed to reinforcement and uniform dispersion of TiC particles, grain size refinement of nickel matrix, stronger interfacial bonding between TiC particles, and nickel matrix. It is clear from the present investigation that TiC reinforcement plays an important role in microstructures as well as microhardness and tribological behavior of Ni-TiC composites. The Ni-TiC metal matrix composites displayed high microhardness and a low coefficient of friction than a pure nickel. Mechanical alloying followed by spark plasma sintering process is a feasible way of processing these composites with improved densification and mechanical behavior without any excess grain growth observed in other conventional methods.

## Figures and Tables

**Table 1:** SPS parameters, grain size, and relative density of nickel for Pure Ni, Ni-5TiC, Ni-10TiC, Ni-25TiC, Ni-30TiC, Ni-40TiC, and Ni-50TiC composites.

No.	Specimen ID	Max. Temperature (°C)	Holding Time (min)	Pressure (MPa)	Grain Size Ni	Relative Density
1	Pure Ni	900	5	65	33.67 μm	98.20%
2	Ni-5TiC	900	5	65	0.43 μm	96.02%
3	Ni-10TiC	900	5	65	0.36 μm	93.39%
4	Ni-25TiC	900	5	65	0.12 μm	86.01%
5	Ni-30TiC	900	5	65	0.11 μm	84.01%
6	Ni-30TiC	1200	5	65	0.21 μm	98.27%
7	Ni-40TiC	1200	5	65	0.20 μm	94.46%
8	Ni-50TiC	1200	5	65	0.19 μm	91.77%

**Table 2:** Mechanical properties of Ni-TiC composites.

Samples and Sintering Temperature	Yield Strength $\sigma_{0.2}$ (MPa)	Compressive Strength $\sigma_{UCS}$ (MPa)	Compressive Strain $\varepsilon_{c}$ (%)	Hardness (HV)	Young’s Modulus (MPa)
Ni-5TiC-900 °C	1393.72	1414.01	4.93	294.7	29,735.41
Ni-10TiC-900 °C	1492.54	1511.27	5.79	338.4	27,351.03
Ni-25TiC-900 °C	1199.5	1202.52	4.14	483.1	29,287.06
Ni-30TiC-900 °C	97.55	288.02	2.99	549	17,573.48
Ni-30TiC-1200 °C	1436	1585.26	6.10	601.4	29,914.28
Ni-40TiC-1200 °C	1166.18	1175.44	4.35	743.9	26,993.81
Ni-50TiC-1200 °C	1390.06	1413.21	5.42	877.8	26,360.78
